# Cluster analysis of hemorrhagic disease in Missouri’s white-tailed deer population: 1980–2013

**DOI:** 10.1186/s12898-018-0188-6

**Published:** 2018-09-14

**Authors:** Gerry Baygents, Majid Bani-Yaghoub

**Affiliations:** 1 Trinidad State Junior College, Valley Campus, 1011 Main Street, Alamosa, CO 81101 USA; 2 Department of Math and Statistics, University of Missouri-Kansas City, 5120 Rockhill Road, Kansas City, MO 64110 USA

**Keywords:** Cluster analysis, *Culicoide*s midges, Hemorrhagic disease, Missouri, SaTScan, White-tailed deer

## Abstract

**Background:**

Outbreaks of deer hemorrhagic disease (HD) have been documented in the USA for many decades. In the year 2012, there was a severe HD outbreak in Missouri with mortalities reaching approximately 6.9 per thousand. Moreover, Missouri accounted for more than 43% of all reported epizootic HD cases in captive white-tailed deer. Using the data of suspected HD occurrence in Missouri, the primary goal of this paper was to determine if HD in Missouri’s white-tailed deer occurs in spatial clusters.

**Results:**

The main results of the cluster analysis are as follows. First, the spatial clusters of years 1980, 1988, 2005–2007, 2010, 2012, and 2013 suggest patterns of outbreaks every 6–8 years, with a potential outbreak in years 2018–2020. Secondly, these spatial clusters were more frequent in the central and southern counties.

**Conclusions:**

The clustering analyses employed in this study have potential applications for improving surveillance programs and designing early warning systems for effective deer population management and potentially reducing the number of HD cases.

## Background

Epizootic hemorrhagic disease (EHD) is an often-fatal hemorrhagic disease of white-tailed deer (*Odocoileus virginianus*) and other ruminants. EHD is vectored to mammals by tiny biting flies, the most well-documented in North America being the *Culicoides* midges [1]. In addition, the bluetongue virus has also been a major issue in white-tailed deer population [2, 3]. Because symptoms caused by EHD and bluetongue are nearly indistinguishable, they are frequently grouped together and referred to as hemorrhagic disease (HD), and the first suspected outbreak of HD in the USA occurred in the 1890s [4].

There are three different expressions of hemorrhagic diseases: peracute, acute, and chronic. The peracute form is the most aggressive and it can cause death within a week. The clinical signs of peracute HD include swelling in the head, tongue, neck, and lungs due to fluid accumulation [5]. The acute HD causes death within 1–2 weeks. Symptoms include swelling and hemorrhage throughout the body, sloughing of hooves, and may include sores or ulcers to form on the deer’s tongue, on portions of the stomach, and on the roof of the mouth [4]. The chronic form of HD consists of nearly 15% of the cases, in which the infected deer will survive with some degree of tissue damage [6]. Secondary infections may lead to death, but if female deer survives, she will pass on antibodies to the HD virus to her offspring [7].

There have been previous studies of the transmission and spread of HD throughout the southeastern United States. Briefly, there is a correlation between the number of HD cases and the number of deer in a population with the virus, and there is strong evidence that the maximum number of cases occurs at intermediate levels (~ 50%) of this seroprevalence. Moreover, there is further support that the relationship between levels of seroprevalence and the number of cases reported is both non-monotonic (with a local minimum ~ 25%) and unimodal [8]. However, management actions to reduce or eliminate HD outbreaks are elusive [9]. One problem is that experimental tests of management treatments are not practical unless one can reasonably predict the locations of HD outbreaks. Models that could predict outbreaks of HD could allow tests of the efficacy of proposed management actions (e.g., supplemental water and fencing of ponds from livestock and wildlife).

Spatial and temporal patterns of HD have been described in the southeast United States by using the space–time K function and Martin Kuldorff’s scan statistic [10–13]. Significant clusters were most evident in Alabama, North Carolina, and South Carolina between 1980 and 2013. Other studies have applied Kulldorff’s space and space–time scan statistic to several geographical regions affected by various disease outbreaks [14–18]. Over 43% of US cases of all 2012 reported EHD cases in captive white-tailed deer belonged to the State of Missouri (see Table 3 of [19]), and, in a previous study, Beringer [5] noted that the HD exposure rate could be as high as 24% within Missouri’s white-tailed deer population. Moreover, there have been four major HD outbreaks in Missouri’s white-tailed deer population in years 1988, 2005, 2007 and 2012. Therefore, there is a need to further investigate the HD dynamics in Missouri. The most severe outbreak was in the year 2012 when every county in Missouri reported at least one case of HD with more than 10,000 cases of mortality. The primary goals of this paper are to identify spatial patterns of HD outbreaks and to statistically determine if HD in Missouri’s white-tailed deer occurs in space and time clusters. This study can be of particular interest to the Missouri Department of Conservation (MDC) as well as cattle and white-tailed deer breeders in the state of Missouri.

## Methods

The MDC provided data on the size and location of deer population and the number of suspected HD occurrences in the wild (and not captured deer data). We note that only a small percentage of the data was actually confirmed as HD due to the time constraints of viable testing after death. The remaining portion of the data was collected by MDC officials based on observed symptoms. Estimated instances of HD, by county, were available for the years 1980, 1988, 2005–2007, 2010, 2012, and 2013. Estimates of deer population were available for all years except 1980, 1988, and 2013. In order to apply Kulldorff’s spatial and space–time scan statistics to the data, we used SaTScan version 9.4.2 [20] over the 33-year study period. The geographic center (centroid) of each county was used to represent the location of the presence of (or absence of) HD in the county.

Kulldorff’s space and space–time scan statistics [21, 22] use a theoretical cylindrical window with a circular (or elliptical) base. The base is geographic and, in turn, is centered on each of several possible grid points throughout the area of study. For each grid point, the radius of the window varies continuously in size from zero to a user-specified upper limit based on distance and/or percentage of population. The height of the cylinder corresponds to a period of time within the study period. These cylindrical windows vary in space and/or in time. Thus, for each possible geographic location, it considers multiple-sized circles around the location and multiple possible time frames. For each location and scanning window, the program computes a likelihood ratio based on the number of observed cases versus the number of expected cases both inside and outside the window, using different probability models depending on the data. This expected value is determined by a user-defined number of replications of the data. The number of incidents remains the same, but their distribution in the region is random. The program determines the significance of a cluster based on the actual number of incidents in each window in comparison to the expected number of incidents based on all the replications. With the discrete Poisson model, the program and analysis assumes that the number of cases at each location follows a Poisson distribution and that the expected number of cases in each location is proportional to its population size. The space–time permutation model requires only case data and the number of observed cases in a cluster is compared to what would have been expected if all cases were independent of each other in both space and time as if there were no space–time interaction. Under the null hypothesis of no significant clusters in the window, the window with the largest likelihood statistic is the most likely cluster. The program also identifies all secondary clusters with a *P* value less than 0.05.

We used three different scans within SaTScan version 9.4. First, for the spatial scan statistic, we used the annual data to locate clusters in each year and to observe how these clusters changed across years. Second, the space–time scan statistic was used. The space–time permutation model is ideal because it requires only case data, with information about the spatial location and time for each case. Moreover, it has the potential of identifying clusters that may not have been significant for any one specific year but are over spans of multiple years. Third, the spatial scan with temporal trends was applied to all cases over the study period to locate clusters with more significant variations in the percentage change in the number of cases per year. As part of the scan analysis, we chose elliptical scanning windows. For the grid points, we used the centroid of each county. When SaTScan identified a centroid within a cluster, we assumed the entire county was within the cluster. In cases where part of a country was within a particular ellipse, those counties were not included in the cluster if the centroid of the county was not included. We set the maximum spatial window to 50% of the total population, the maximum temporal window (when needed) to 50% of the study period with a 1-calendar year time aggregation to locate fewer, larger clusters. The number of random Monte Carlo replications to 4999. For the years when population data was not available, SaTScan estimated the population through linear interpolation. No additional information about controls or background population at risk is necessary.

## Results

### Data analysis

There were 16,853 cases of suspected HD reports over all 114 Missouri counties during the study period. If we count the number of times each county reported at least one case, there were 406 times a county reported at least one case (out of 912 potential reporting times). During all years represented, 2012 had the largest number of cases (10,177) with all counties reporting at least one case and the estimated prevalence of 6.9 deer per thousand. Table 1 provides a summary of deer population, HD incidents, the number of counties affected and prevalence per thousand.

**Table 1:** A summary of the estimated deer population in Missouri, the number of counties (out of 114) reporting suspected HD cases, the number of suspected HD incidents, and the prevalence of suspected HD in thousands

Year	1980	1988	2005	2006	2007	2010	2012	2013
Harvest	53,298	149,064	286,027	321,828	298,360	272,534	307,979	250,135
Population^a^	NA	NA	1,490,491	1,506,568	1,420,170	1,433,966	1,475,126	NA
Counties	41	71	21	13	91	2	114	43
Incidents	315	1410	772	484	3095	150	10,177	450
Prevalence^b^	NA	NA	0.517	0.321	2.179	0.105	6.899	NA

^a^Estimated deer population and therefore the prevalences were not available for the years 1980, 1988 and 2013

^b^The estimated prevalence is per thousand

### Spatial clusters by individual years

**Fig. 1:**
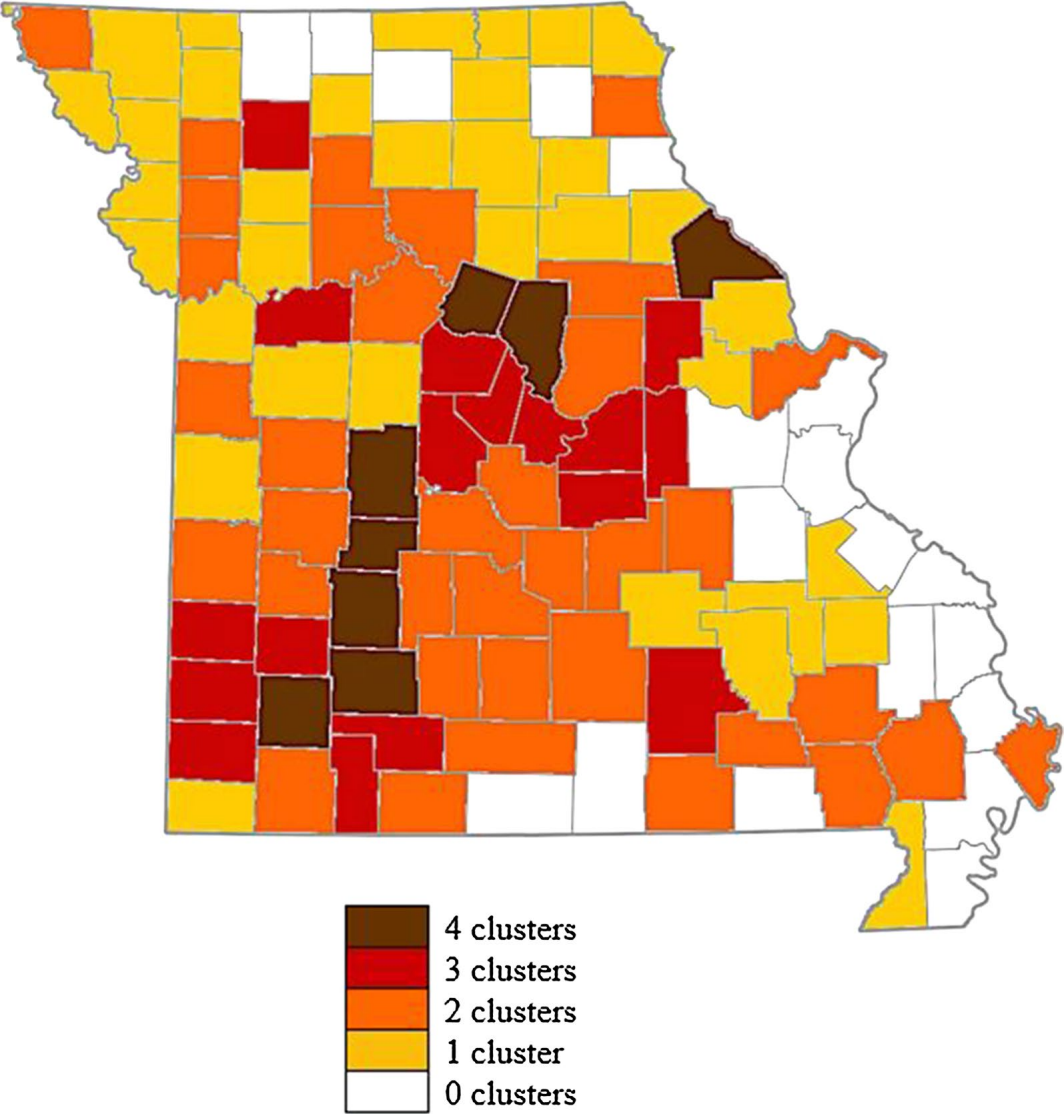
Frequency (in number of years) of HD cluster occurrence for each county during the study period. The darker the shading, the more frequently it was identified in a cluster

**Fig. 2:**
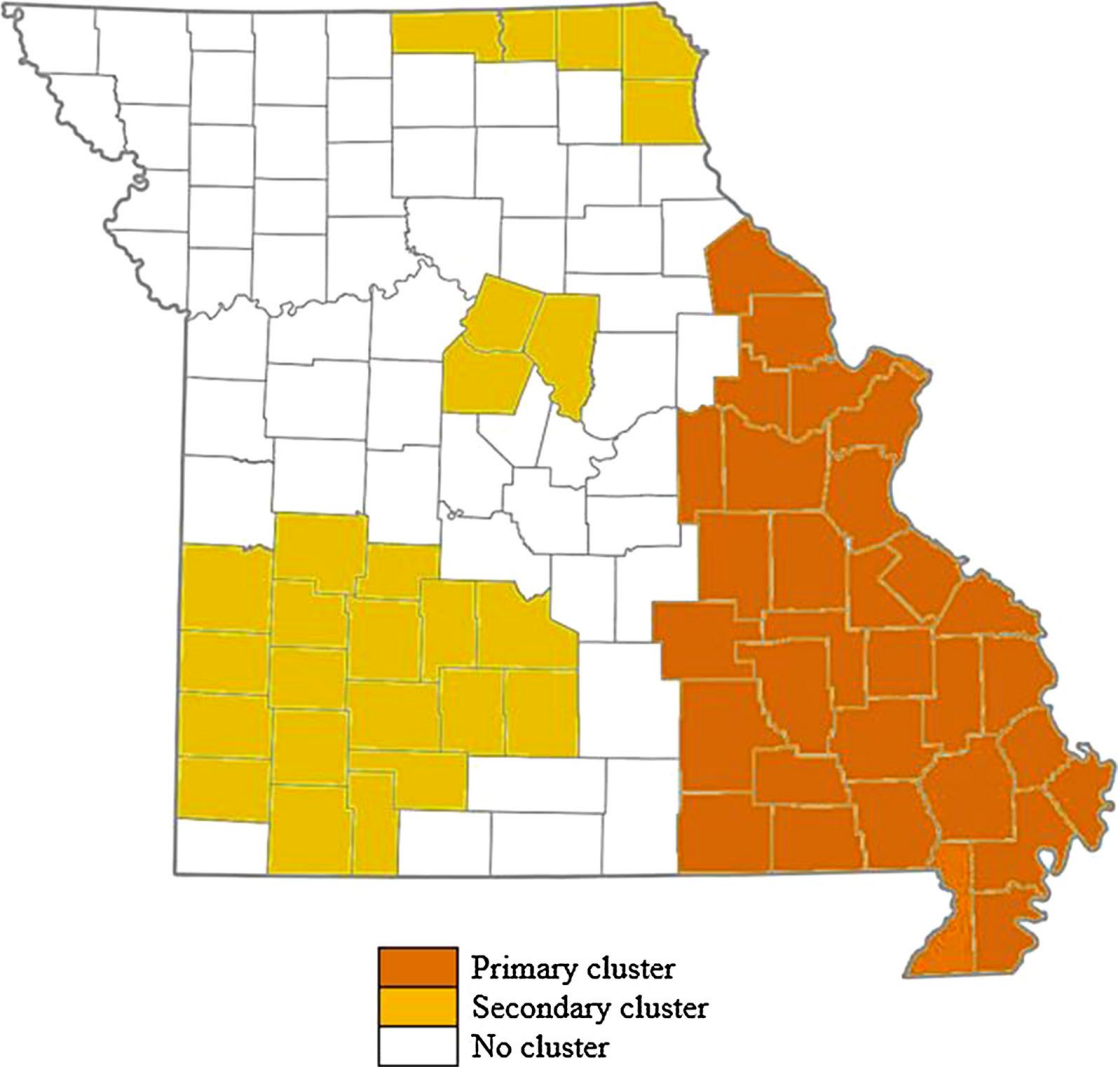
Spatial cluster of years 1980–2013 suggests presence of 6–8 years cycles of HD outbreaks in Missouri. An HD outbreak is anticipated for during 2018–2020

Table 2 provides the locations of the most significant cluster in each year that HD data was available. In Fig. 1, counties are shaded based on the number of years in which SaTScan identified them as part of any cluster (primary or secondary) within any one singular year during the entire study period. The darker the shading, the more frequently it was identified. We observe that SaTScan identified clusters in central to southwestern Missouri more frequently. Figure 2 shows primary and secondary clusters over the study period. Although there is a gap between 1988 and 2005 data, we can see that the outbreaks have occurred in cycles of 6–8 years.

**Table 2:** The most significant spatial clusters of HD (by individual year) in white-tailed deer during the entire study period (1980–2013) with a maximum spatial window = 50% of the total population

Year	Location(s)^a^	Observed	Expected	*P*-value
1980	Central (Howard)	189	15.52	*P* < 0.0001
1988	Central, South (Laclede)	762	337.18	*P* < 0.0001
2005	Central, Southwest (Dallas)	711	263.86	*P* < 0.0001
2006	Southwest (Dade)	246	42.64	*P* < 0.0001
2007	East (Lincoln)	843	91.31	*P* < 0.0001
2010	Central (Saline)	150	3.11	*P* < 0.0001
2012	Central, West (Lafayette)	5358	3223.70	*P* < 0.0001
2013	Northeast (Clark)	270	20.94	*P* < 0.0001

^a^Denotes county of approximate center of each cluster. Observed: the number of incidents in the most significant cluster only. Expected: the expected number of incidents in the cluster based on the random replications. The number of estimated HD cases was available only for the years presented here

### Spatio-temporal clusters

**Fig. 3:**
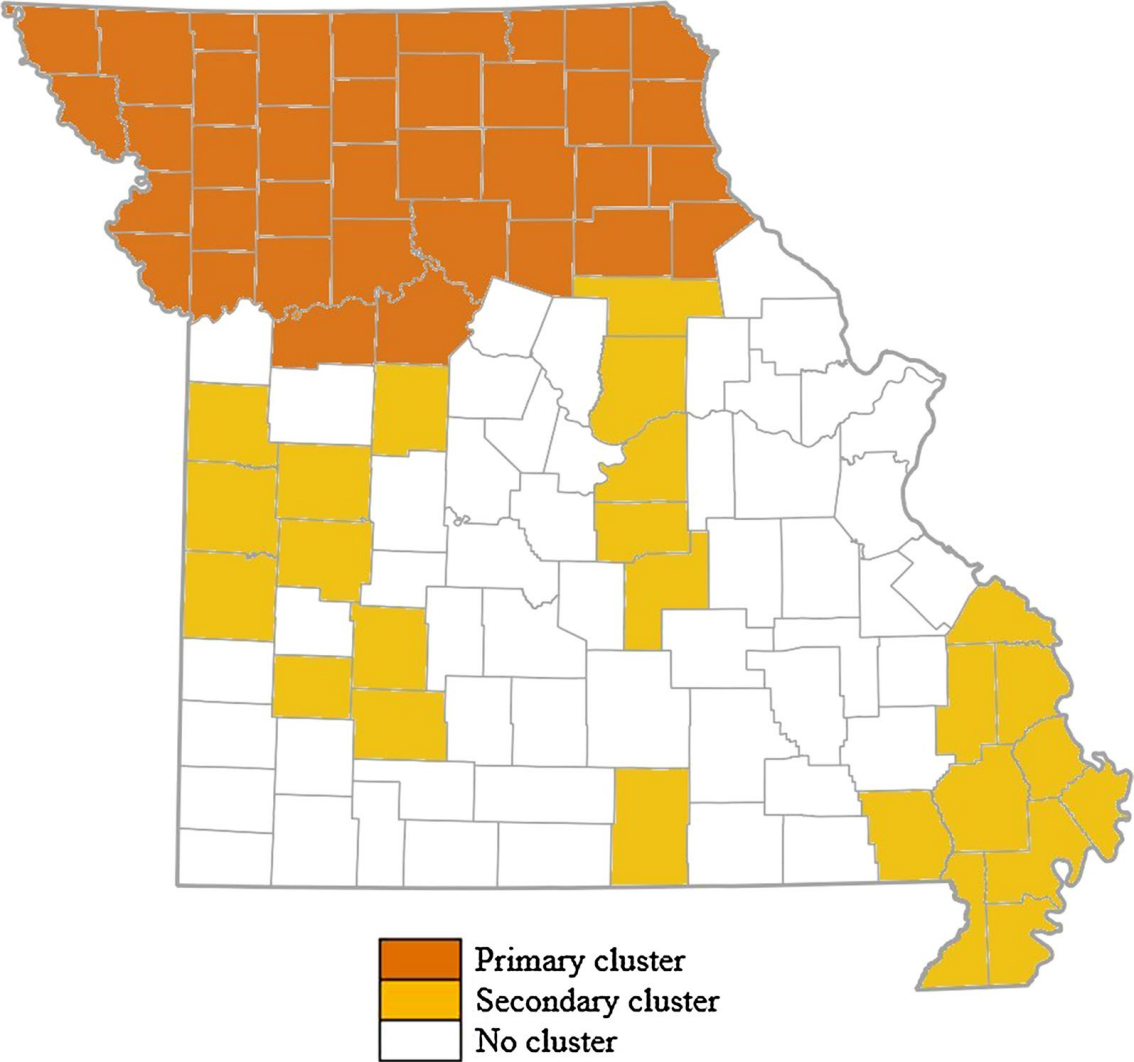
Significant spatio-temporal cluster of HD in white-tailed deer (1980–2013). Primary and secondary clusters of HD presence are displayed as orange and yellow, respectively

Four significant spatio-temporal clusters were detected, where the primary cluster consists of 32 counties in the eastern and southeastern portions of Missouri. Figure 3 shows the locations of the significant spatio-temporal primary and secondary clusters. The three secondary clusters were located in the southwest (cluster 2), a small portion in the northeast (cluster 3), and a small cluster in the center of the state (cluster 4). See Table 3 for a summary of the significant clusters and the number of counties affected.

**Table 3:** Significant spatial–temporal clusters of HD in white-tailed deer during the entire study period (1980–2013) with maximum spatial window = 50% of the total number of HD cases and maximum temporal window = 50% of the entire study period

	Cluster	Counties	Observed	Expected	*P*-value	Period
Primary	1	32	1993	785.95	*P *< 0.0001	2006–2007
Secondary	2	18	657	168.66	*P *< 0.0001	2005–2006
	3	5	270	20.69	*P *< 0.0001	2013
	4	3	160	11.45	*P *< 0.0001	1980

Cluster: cluster ID. Counties: the number of counties in each cluster. Observed: the number of incidents in each cluster. Expected: the expected number of incidents in the cluster based on the random replications

### Temporal trends in spatial clusters

**Fig. 4:**
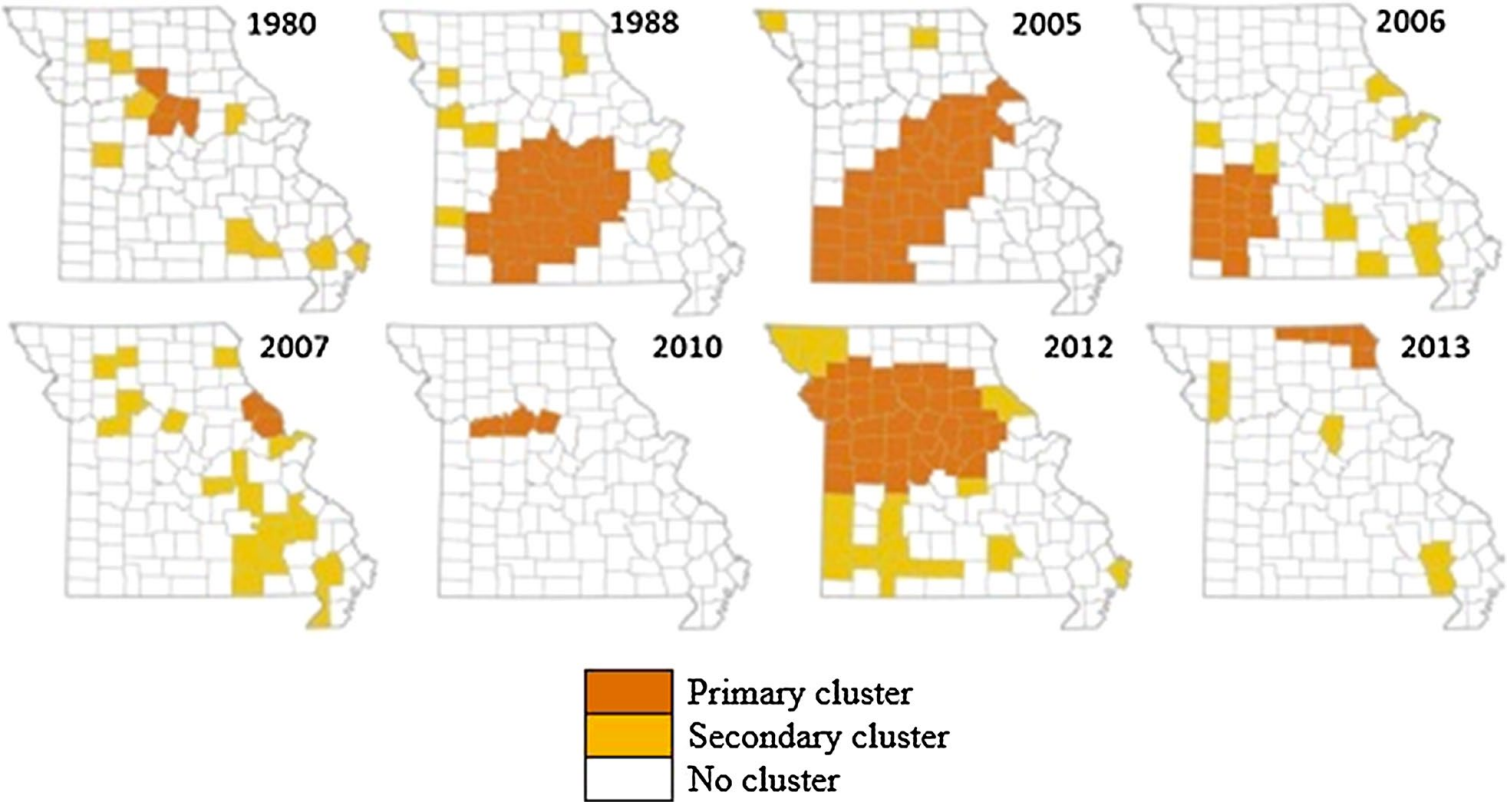
Significant temporal trends (annual increases) of HD in white-tailed deer (1980–2013). Primary and secondary clusters of HD presence are displayed as orange and yellow, respectively. In the cases where a secondary cluster overlaps the primary cluster, the counties in the overlap are grouped within the primary cluster

A trend of 19% annual increase was detected over the study period. There were no instances where a cluster had a significant annual decrease, and Fig. 4 shows where the annual increase was the most significant. The primary cluster is the northernmost third of Missouri. In the cases where a secondary cluster overlaps the primary cluster, the counties in the overlap are grouped within the primary cluster. Table 4 gives the proportion of cases in each cluster and its trend of annual increase. The highest trend of annual increase belongs to Howell County in southern Missouri. However, the five counties (Audrain, Calla way, Osage, Maries, and Phelps) in central Missouri have the highest number of annual cases (57.6 per 100,000).

**Table 4:** Significant temporal trends of hemorrhagic disease in white-tailed deer during the entire study period (1980–2013) with maximum spatial window = 50% of the total number of HD cases and maximum temporal window = 50% of the entire study period

	Cluster	Counties	Location (Fig. 2)	Annual cases (per 100,000)	Trend of annual increase (%)	*P*-value
Primary	1	37	North	33.1	32.1	0.0002
Secondary	2	5	Central	57.6	30.9	0.0002
	3	4	West	46.6	31.1	0.0002
	4	10	Southeast	15.4	33.2	0.0002
	5	3	West	21.0	31.3	0.0002
	6	2	Southwest	21.6	38.5	0.0006
	7	1	South	2.7	102.1	0.0018

Cluster: cluster ID. Counties: the number of counties in each cluster. Observed: the number of incidents in each cluster

## Conclusions

In summary, using the statistical models and the available data, we identified the significant spatial and the spatiotemporal clusters of HD in white-tailed deer population residing in Missouri. The most significant spatiotemporal cluster was identified in the southeastern counties of Missouri (see Fig. 2), and the most significant temporal trend was identified in the northern counties (see Fig. 3). These trends and clusters are in agreement with the density of captive white-tailed deer EHD cases during the most severe outbreak in 2012 (see Figure 3 of [19]). However, as shown in Fig. 1, the frequencies of significant spatial clusters are mainly located in the central and southwestern counties. Thus, there is a greater likelihood of outbreaks in the central and southwestern counties. Moreover, the spatial clusters shown in Fig. 2 suggest that there might be patterns of HD outbreaks. Xu et al. [23] identified similar cycles of 6–8 years in an independent study of HD outbreak in the southeastern USA. Therefore, we speculate that there will be an HD outbreak in Missouri’s white-tailed deer population between the years 2018–2020.

## Discussion

It is important to note that HD occurs seasonally and nearly all reported cases occur during late summer and fall. This seasonal occurrence could be related to high abundance of *Culicoides* biting midges during late summer and fall as they transmit the disease. In particular, it is likely that HD outbreaks are more prevalent when weather conditions during the late summer and fall cause an abundance of muddy areas where midges breed. This could be due to high summer temperatures that cause bodies of water to recede and leave mud flats or by overly rainy and wet conditions in late spring. Those very rare HD cases that are in late fall and winter represent the chronic form of HD.

As outlined below, this study carries a number of limitations related to the data. In general, data availability in wildlife is often an issue. Populations are not enclosed nor controlled, and getting accurate population counts is impossible. Counting the number of HD occurrences depends on observations of harvested deer. Variations in deer population density, regulations on who may harvest the deer, regulation on how many deer may be harvested, and other factors affect this count. Indirect reports from the public may not be verifiable, and some regions may be restricted to hunters and the public at large. So, in actuality, these reports are only estimations and suspected reports. Also, HD often has a localized effect on the landscape. For example, the vast majority of the reports in Benton County (in western Missouri) were only from the northern half of the county. Furthermore, in years when there is not a significant known outbreak, results were reported to the MDC in January of the following year (if at all), and because of this time lag, there is some concern over the accuracy of the reports. Regardless, information of the spatiotemporal clustering may improve or design local surveillance and early warning systems [24, 25]. In particular, areas with spatial and spatiotemporal HD clusters can be targets of more frequent surveillance. These programs can serve as a sentinel to reduce number of HD cases in local farms and to sustain free-living deer population.

Currently there are no effective wildlife management tools or strategies to control or prevent the hemorrhagic diseases in wildlife [6]. However, fencing off livestock and captive white-tailed deer from ponds can reduce the probability of encountering midges. Thus, conservationists and wildlife managers may be able to use the outcomes of the clustering analyses to establish an early warning system to reduce the number of HD cases in livestock and captive white-tailed deer. An early warning system is also necessary for correct management of the free-living deer population. In particular, an early detection of HD outbreak can critically help the MDC officials to reduce the number of hunting permits in order to sustain the deer population in subsequent seasons. The outcomes of the clustering analysis provided in this study reveals the significant magnitudes and directions of the HD spread in Missouri in the past three decades. In conclusion, cluster analyses can improve our understanding of the epidemiology of hemorrhagic diseases and it can lead to designing effective surveillance and early warning programs.

The Missouri Department of Conservation provided the data used in this analysis. The authors are also thankful to multiple reviewers, including Dr. Aaron Reed at the School of Biological Sciences at UMKC, for valuable suggestions to improve the readability and quality of both this paper and this research.

## Abbreviations

EHD: epizootic hemorrhagic disease
HD: hemorrhagic disease
MDC: Missouri Department of Conservation

## References

[1] Nettles VF, Stallknecht DE (1992). History and progress in the study of hemorrhagic disease of deer. Trans N Am Wildl Nat Resour.

[2] Wieser-Schimpf L, Wilson WC, French DD, Baham A, Foil LD (1993). Bluetongue virus in sheep and cattle and *Culicoides variipennis* and *C. stellifer* (Diptera: Ceratopogonidae) in Louisiana. J Med Entomol.

[3] Stallknecht DE, Luttrell MP, Smith KE, Nettles VF (1996). Hemorrhagic disease in white-tailed deer in Texas: a case for enzootic stability. J Wildl Dis.

[4] Hoff G, Trainer DO, Davis JW, Karstad LH, Trainer DO (1981). Hemorrhagic disease in wild ruminants. Infectious diseases of wild mammals.

[5] Beringer J, Hansen LP, Stallknecht DE (2000). An epizootic of hemorrhagic disease in white-tailed deer in Missouri. J Wildl Dis.

[6] Flinn E, Sumners J (2013). Breaking down the hemorrhagic disease outbreak. Mo Conserv.

[7] Flinn E, Sumners J (2013). State of the state’s deer herd. Mo Conserv.

[8] Park AW, Magori K, White BA, Stallknecht DE (2013). When more transmission equals less disease: reconciling the disconnect between disease hotspots and parasite transmission. PLoS ONE.

[9] Pfannensteil RS, Mullens BA, Ruder MG, Zurek L, Cohnstaedt LW, Nayduch D (2015). Management of North American *Culicoides* biting midges: current knowledge and research needs. Vector Borne Zoonotic Dis.

[10] Diggle P, Chetwynd AG, Haggkvist R, Morris SE (1995). Second-order analysis of space–time clustering. Stat Methods Med Res.

[11] Kulldorff M, Athas WF, Feuer EJ, Miller BA, Key CR (1998). Evaluating cluster alarms: a space–time scan statistic and brain cancer in Los Alamos, New Mexico. Am J Public Health.

[12] Song C, Kulldorff M (2003). Power evaluation of disease clustering tests. Int J Health Geogr.

[13] Hwang S. Extending spatial hot spot detection techniques to temporal dimensions. In: Proceedings of the 4th ISPRS workshop on dynamic and multi-dimensional GIS, University of Glamorgan, Wales, UK, September, 2005. p. 5–8.

[14] Norström M, Pfeiffer DU, Jarp J (2000). A space–time cluster investigation of an outbreak of acute respiratory disease in Norwegian cattle herds. Prev Vet Med.

[15] Ward MP (2002). Clustering of reported cases of leptospirosis among dogs in the United States and Canada. Prev Vet Med.

[16] Leblond A, Sandoz A, Lefebvre G, Zeller H, Bicout DJ (2007). Remote sensing based identification of environmental risk factors associated with West Nile disease in horses in Camargue, France. Prev Vet Med.

[17] Gautam R, Srinath I, Clavijo A, Szonyi B, Bani-Yaghou M, Park S, Ivanek R (2013). Identifying areas of high risk of human exposure to coccidioidomycosis in Texas using serology data from dogs. Zoonoses Public Health.

[18] Mirghani SE, Nour BY, Bushra SM, Elhassan IM, Snow RW, Noor AM (2010). The spatial–temporal clustering of *Plasmodium falciparum* infection over eleven years in Gezira State, The Sudan. Malar J.

[19] Stevens G, McCluskey B, King A, O’Hearn E, Mayr G (2015). Review of the 2012 epizootic hemorrhagic disease outbreak in domestic ruminants in the United States. PLoS ONE.

[20] Kulldorff M. SatScan user guide for version 9.4. 2006. http://www.satscan.org/.

[21] Kulldorff M (1997). A spatial scan statistic. Commun Stat Theor Methods.

[22] Kulldorff M, Heffernan R, Hartman J, Assunção RM, Mostashari F (2005). A space–time permutation scan statistic for the early detection of disease outbreaks. PLoS Med.

[23] Xu B, Madden M, Stallknecht D, Hodler T, Parker K (2012). Spatial and spatial–temporal clustering analysis of hemorrhagic diseases in white-tailed deer in the southeastern USA: 1980–2003. Prev Vet Med.

[24] Stallknecht DE, Howerth EW (2004). Epidemiology of bluetongue and epizootic hemorrhagic disease in wildlife: surveillance methods. Vet Ital.

[25] Nettles VF, Davidson WR, Stallknecht DE. Surveillance for hemorrhagic disease in white-tailed deer and other wild ruminants, 1980–1989. In: Proceedings of annual conference of southeastern association of fish and wildlife agencies. 1992. pp. 138–146.

